# Global Wheat Head Detection Challenges: Winning Models and Application for Head Counting

**DOI:** 10.34133/plantphenomics.0059

**Published:** 2023-06-26

**Authors:** Etienne David, Franklin Ogidi, Daniel Smith, Scott Chapman, Benoit de Solan, Wei Guo, Frederic Baret, Ian Stavness

**Affiliations:** ^1^ UMR 1114 EMMAH, INRAE, Avignon, France.; ^2^ Arvalis – Institut du Végétal, Paris, France.; ^3^ Department of Computer Science, University of Saskatchewan, Saskatoon, Canada.; ^4^ School of Food and Agricultural Sciences, University of Queensland, Brisbane, Australia.; ^5^ Graduate School of Agricultural and Life Sciences, The University of Tokyo, Tokyo, Japan.

## Abstract

Data competitions have become a popular approach to crowdsource new data analysis methods for general and specialized data science problems. Data competitions have a rich history in plant phenotyping, and new outdoor field datasets have the potential to embrace solutions across research and commercial applications. We developed the Global Wheat Challenge as a generalization competition in 2020 and 2021 to find more robust solutions for wheat head detection using field images from different regions. We analyze the winning challenge solutions in terms of their robustness when applied to new datasets. We found that the design of the competition had an influence on the selection of winning solutions and provide recommendations for future competitions to encourage the selection of more robust solutions.

## Introduction

Crowdsourcing is an increasingly popular approach for scientists to make advances in their field by collecting diverse raw or labeled data [1–4], solving problems that are difficult for algorithms but easy for humans, such as protein folding [5], or accessing large-scale distributed computing power [6]. Crowdsourcing of data analysis has increased rapidly in recent years due to the popularity of Big Data challenges [7] on web platforms such as Kaggle, Codalab, and AIcrowd. In particular, problems that are amenable to machine learning (ML) approaches such as computer vision problems have been promoted and popularized through open competitions such as ImageNet or COCO [8,9].

Crowdsourcing of data annotation and analysis have also expanded to specific application areas, such as image-based plant phenotyping, where deep learning methods have been employed for plant disease classification [10–12], plant and organ detection and counting [13–16], and vegetation segmentation [17]. In this context, crowdsourcing of data and analysis helps to connect domain experts, e.g., plant scientists, with computer and data scientists. Such collaboration with data scientists outside the traditional scope of plant phenotyping is essential to solve fundamental data science problems within the domain.

Early computer vision competitions for plant phenotyping problems [18,19] such as leaf counting [20] and leaf segmentation [18] were highly successful. Plant phenotyping competitions have typically focused on indoor, controlled single plant images although a few recent competitions have included outdoor field data such as the Agriculture-Vision competition [21] or the GrassClover competition [22]. Expanding data competition to agricultural applications is crucial for facing the new challenges of global food production in the context of global change. The availability of sensors, vectors, and data for agricultural applications is rapidly expanding, while effective data interpretation pipelines are still limited. Therefore, crowdsourcing new approaches could advance the data interpretation challenge in plant phenotyping and agriculture and foster the deployment of ML cameras on farming equipment.

Despite the use of deep learning in recent plant phenotyping studies, the robustness and generalizability of these methods remains an open question, particularly for small plant datasets. Most deep learning algorithms require a large set of labeled images to be trained on, such as ImageNet [8] or MS COCO [9]. The process of labeling such images is expensive and tedious, limiting the availability of large training datasets. A large diversity of examples within a dataset is also important to study robustness of trained models. Such large and diverse datasets are already available for indoor plant phenotyping [20,23]. However, under field conditions, the lack of sufficiently large and diverse real-world datasets [24,25] prevents from evaluating the robustness of the trained plant phenotyping models.

Detecting wheat heads in field conditions is desired by breeders and agronomists, because it allows estimating the head density, which is one of the main yield components for wheat. Wheat head detection also allows for the localization of plants and descriptions of head emergence over time and space, which has consequences for plant competition and microplot heterogeneity. Finally, detection is the first step before further characterization of wheat heads, e.g., to examine size, health, or disease of heads and spikelets. Several studies proposed successful methods from high-resolution RGB (red-green-blue) images and deep learning methods [13,26]. However, the training and testing datasets in these studies were limited to individual wheat fields, and it is unclear if their results apply to new datasets because the possible variations in sensors, illumination conditions, development stages, and genotypes could impact the models performance [25].

We compiled the Global Wheat Head Dataset (GWHD) [14,27] to study the robustness problem in plant phenotyping. The GWHD is a large and comprehensive labeled dataset for wheat head localization. Based on the GHWD, we organized 2 challenges to attract a large cohort of ML practitioners to solve the wheat head detection problem: the Global Wheat Challenge 2020 (GWC_2020) on Kaggle, which took place from in 2020 and attracted 2,245 competitors and the Global Wheat Challenge 2021 (GWC_2021) on AIcrowd, which took place in 2021 and attracted 432 competitors. This paper summarizes these competitions, including a description of the most successful approaches proposed. We further evaluate their robustness on 2 new test datasets. Finally, we discuss lessons learned from this competition and provide recommendations for future ones. In particular, advices on how to organize a competition are provided in Section S4.

## Materials and Methods

### Datasets

Three datasets are used in this study (Table 1): (a) the GWHD, (b), the University of Queensland Frame (UQ Frame) dataset, and (c) the Arvalis LITERAL (LITE phenotyping system to Record, Analyze and Lay out) dataset. Image annotations consisted of a set of bounding boxes around all wheat heads, which is the standard annotation format for training object detection algorithms, such as Faster-RCNN (region-based convolutional neural network) [28]. The GWHD includes the annotated images, whereas the UQ Frame dataset includes both annotated images and in-field counting. The Arvalis LITERAL dataset consists of head counts made manually in the field within a 0.25 × 0.25-m sampling area bounded by a physical frame and the corresponding RGB images that were not annotated.

**Table 1.** Summary of the 3 datasets used in the study.

Datasets	Use	Image annotation	In-field counting	Comments
Global What Head	Training and test	Yes	No	Described in [27]
UQ Frame	Test	Yes	Yes	In-field count are made on the same sampling area as the digital images
Arvalis LITERAL	Test	No	Yes	In-field density is manually evaluated on other parts of the microplots

#### GWHD

The Global Wheat Head Detection dataset used for the competition has been previously described in [14,27]. It contains 6 515 high-resolution RGB images representing 275 187 wheat heads from 16 institutions across 5 continents and 12 countries (Table 2). It contains 47 subdatasets corresponding to an image acquisition session where images are acquired at a single date over a single site with a single camera system. The images were taken from 1 to 2 m from the soil using different platforms (handheld, cart, tractor, gantry, and spidercam) and different high-resolution RGB cameras, providing a ground sampling distance between 0.1 and 0.43 mm. All the images were resized to 1,024 × 1,024 px. Images were acquired at a range of development stages, from postflowering to ripening. The images were carefully labeled by several operators to ensure consistency and reliability. The latest version is openly available on Zenodo (Global Wheat Head Dataset 2021 | Zenodo). More details are given in Table S1 in the Supplementary Materials.

**Table 2.** Description of the different sessions of UQ Frame dataset.

Session name	Date	Location	Latitude (°)	Longitude (°)	Number of microplots	Frame size (m)
Frame_UQ_7	2020 August 31	Gatton	−27.55	152.27	42	0.5 × 1.0
Frame_UQ_8	2020 September 22	Gatton	−27.55	152.27	53	0.5 × 1.0
Frame_UQ_9	2020 October 6	Gatton	−27.55	152.27	17	0.5 × 0.5
Frame_UQ_10	2020 October 9	McAlister	−33.98	116.40	40	0.55 × 0.55
Frame_UQ_11	2020 October 16	Brookstead	−27.75	151.44	33	0.55 × 0.55

#### UQ Frame dataset

**Fig. 1.**
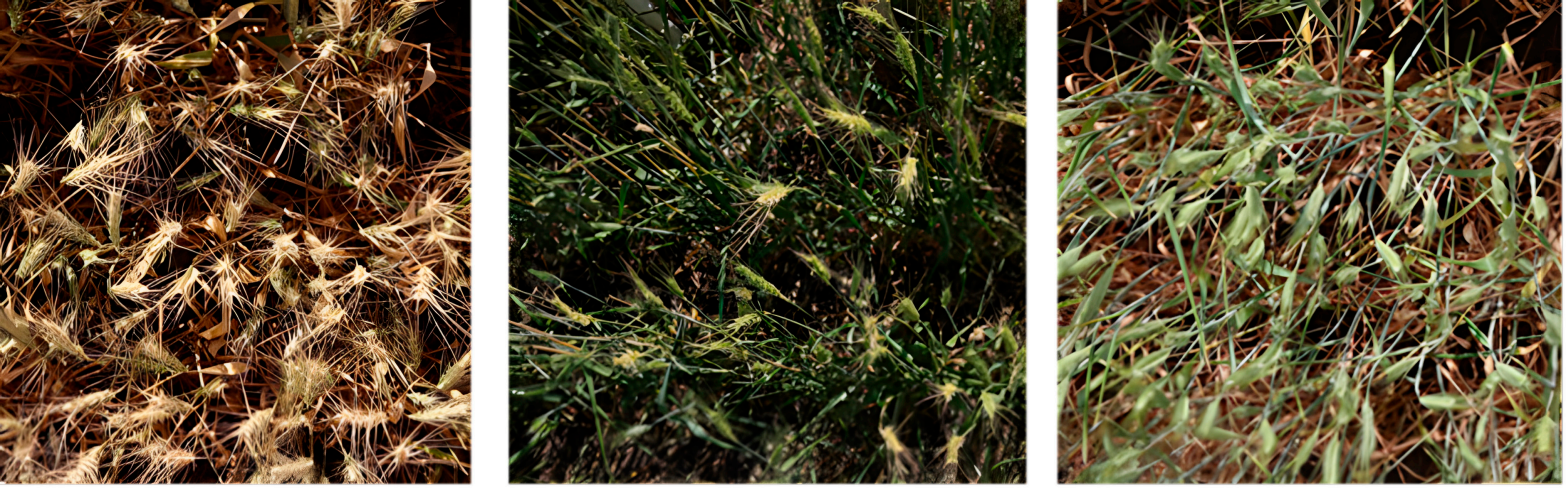
Sample from the UQ Frame dataset.

The UQ Frame dataset is a collection of images acquired on 3 locations across Australia from August to October 2020 and corresponding to 5 sessions (Table 2). For each experiment, a physical frame was placed in the microplot. An RGB image was taken from nadir with a Sony RX0 camera using a handheld system. It provides a ground sampling distance around 0.3 mm. Each image is rotated and cropped to the limit of the frame. Different frames were used and presented in Table 2. Two human operators counted the number of wheat heads within the frame in-field, and the results were averaged. Each corresponding image was also manually labeled with the same methodology as described in [14]. These images were used in the test dataset during the 2021 competition. A sample of the dataset is shown in Fig. 1.

#### Arvalis LITERAL dataset

**Fig. 2.**
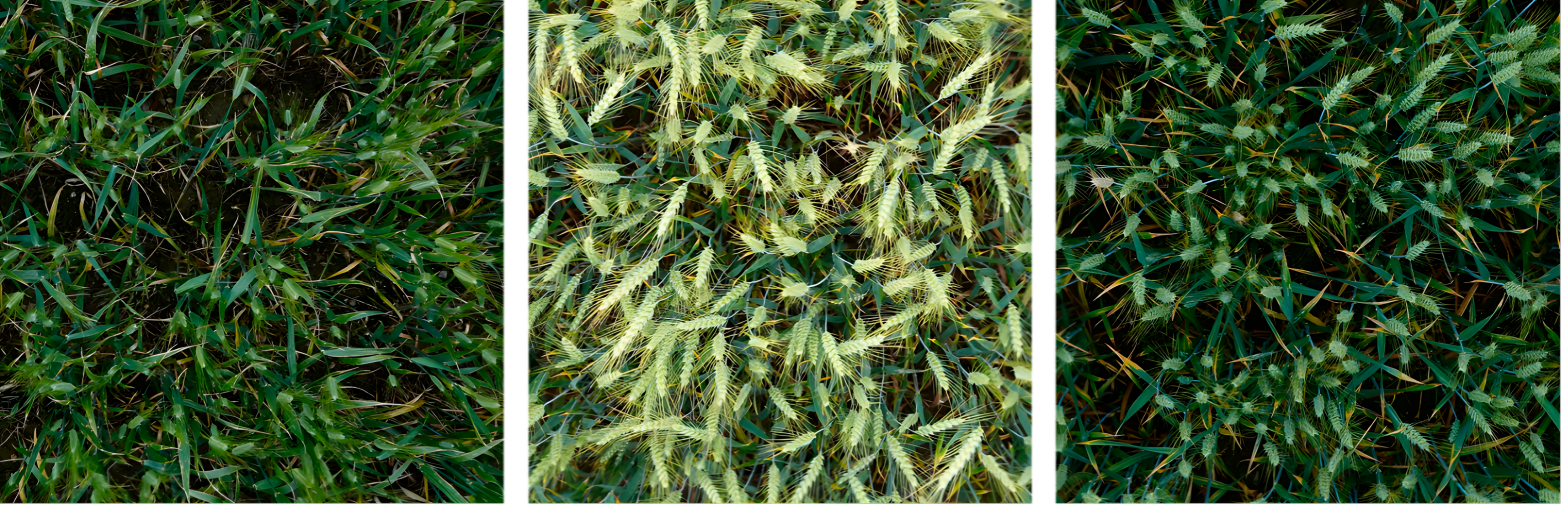
Sample from the Arvalis LITERAL dataset.

The Arvalis LITERAL dataset is a collection of RGB images taken with a handheld system on 5 locations in France, from May to June 2021 and corresponding to a total of 6 sessions (Table 3). Four images per microplot were acquired with Sony RX0 cameras with a resolution of 1,424 × 1,424 px. The camera was at about 2 m from the ground, and the distance between the sensor and the top of the canopy was estimated by stereovision. A sample of the dataset is shown in Fig. 2. The head density for each image was computed as the number of heads in the image divided by the corresponding footprint area derived from the distance to the canopy, the focal length, and the pixel size on the sensor matrix. Finally, the head density per microplot was averaged over the 4 images. Reference in-field head density was measured by counting the heads over 2 adjacent rows of 1-m length segment corresponding to a 0.35-m^2^ sampling area.

**Table 3.** Presentation of the sessions acquired with the LITERAL system.

Session name	Date	Location	Latitude (°)	Longitude (°)	Number of microplots
Greoux	2021 June 18	Gréoux	43.75	5.85	21
OLM_phenohd	2021 June 24	Beauce la Romaine	47.90	1.52	6
OLM_tpgen	2021 June 17	Beauce la Romaine	47.86	1.28	4
Bignan	2021 June 17	Bignan	47.88	−2.75	36
Clermont	2021 June 15	Clermont	45.67	2.86	24
Encrambade	2021 June 4	Encrambade	43.40	1.64	80

### The Global Wheat Challenge 2020 and 2021

The Global Wheat Head Detection 2020 [14] and 2021 [27] datasets were the first datasets designed to match the need for large diversity in imaging conditions, crop state and stage, and wheat genotypes. The 2 challenges organized in 2020 and 2021 were designed to crowdsource the best solutions for wheat head detection. The experience gained with the 2020 challenge allowed to implement improvements on both the dataset and the metrics used to evaluate the solutions. The first challenge took place on Kaggle in 2020 using the GWHD_2020 dataset and gathered 2,245 competitors. The second challenge was held on AIcrowd in 2021 using the GWHD_2021 dataset and attracted 432 participants.

Similar rules were applied to both challenges: the competitors could access the training dataset composed of labeled images from Europe and submit their predictions on a separate dataset consisting of images from North America, Asia, Oceania, and Africa. This set was split into a public set called “validation” and a private set called “test”. Participants could access the images of the validation dataset but not the labels. They could score their solution up to 50 times on this “validation” set. The final ranking was made on the test set. The winning solutions were expected to be open source, with a MIT license for the GWC_2020 and any open-source license allowing unrestricted reuse for the GWC_2021.

The size of the dataset used for the GWC_2021 was growing from 4,700 to 6,515 images corresponding to 9 additional sessions for training and 6 for the private test set. The notion of session, as defined in [27], was introduced to better understand the factors controlling the model robustness: a session is composed of images acquired on a single site, at a single date, with specific illumination conditions and with the same image acquisition system. However, for the original Kaggle competition, the subdatasets were not as clearly defined. The GWC_2021 includes now 47 subdatasets corresponding to measurement sessions. The split between the training, validation, and test datasets was also changed (Table 4). The European sessions used for the training cover all the possible development stages, from first head emergence to maturity. The validation and test sets are entirely disjoint in GWC_2021, while they were randomly drawn from the same subdatasets on GWC_2020.

**Table 4.** Distribution of the subsets between the training, validation, and test datasets for GWC_2020 and GWC_2021.

	Train	Validation (public test)	Test (private test)
GWC_2020	Ethz_1	UQ_1 to UQ_6 ^a^	
	Rres_1	Utokyo_1 to Utokyo_3^a^	
	Arvalis_1 to Arvalis_6	NAU_1^a^	
	Inrae_1		
	Usask_1		
GWC_2021	Ethz_1	UQ_1 to UQ_6	UQ_7 to UQ_11
	Rres_1	Utokyo_1 to Utokyo_3	ARC_1
	ULiège-GxABT_1	NAU_1	Ukyoto_1
	NMBU_1 ; NMBU_2	Usask_1	KSU_1 to KSU_4
	Arvalis_1 to Arvalis_12		Terraref_1 and Terraref_2
	Inrae_1		CIMMYT_1 to CIMMYT_3.

^a^ In 2020, the validation and test sets were randomly sampled (once) from the same subdatasets.

### Metrics used to evaluate the winning solutions

The Intersection over Union (IoU) ratio is used to define the confusion matrix terms. A true positive (TP) is a labeled bounding box that matches a predicted one with an IoU larger than the threshold value. A false positive (FP) is a predicted bounding box having an IoU lower than the threshold with any labeled bounding box. A false negative (FN) is a labeled bounding box having an IoU lower than the threshold with any predicted bounding box. In the case of GWC_2020, the accuracy at the image level, *A_i_*, is the average of the accuracy across IoU values ranging from 0.5 to 0.75 with 0.05 steps.

$$A_{i}=\frac{\mathrm{TP}\left(\mathrm{IoU}\right)}{\mathrm{TP}\left(\mathrm{IoU}\right)+\mathrm{FP}\left(\mathrm{IoU}\right)+\mathrm{FN}\left(\mathrm{IoU}\right)}$$ (1)

Note that the accuracy is also the true positive rate (TPR). Then, the accuracy at the image level was averaged over all the *n* images of the test dataset to get the metric used in the GWC_2020, *AA*_2020_:

$$A_{D}=\frac{1}{n}*\sum_{1}^{n}A_{i},$$ (2)

For GWC_2021, the accuracy is calculated for a unique IoU threshold of 0.5 to favor detection. Further, because the test dataset was composed of several sessions with different size (Tables 1 and 2), we used the average domain accuracy, *AA*_2021_ (Section S1).

$${\mathrm{AA}}_{2021}=\frac{1}{d}\sum_{1}^{d}\frac{1}{n_{D}}*\sum_{1}^{n_{D}}A_{\mathrm{Di}}$$ (3)

where *d* is the number of domains *D*, and *n* the number of images in the domain *D*.

In addition to the metrics presented above for GWC_2021, other metrics were used to further evaluate the possible problems of the solutions proposed: the rates of FP (*FPR* = *FP*/(*TP* + *FP* + *FN*)) and FN (*FNR* = *FN*/(*TP* + *FP* + *FN*)) to better quantify possible detection problems. Further, the root mean square error (RMSE), the relative RMSE (rRMSE), the bias, and the coefficient of determination (*R*^2^) were used to quantify the performances of estimating head counts and head density.

### The models to be compared

We considered the 3 best solutions as ranked using the average domain accuracy defined previously for the GWC_2020 and GWC_2021. Their codes were made publicly available by the participants. Further, we used Faster-RCNN as the baseline solution and implemented as in [13,14] with an input image size of 512 × 512 px. All solutions were trained on the GWC_2021 splits, which means that the GWC_2020 solutions were retrained from scratch from the provided code. The Faster-RCNN was trained during 30 epochs on a Nvidia GeForce 3090 RTX with 24GB of RAM. The baseline and winning solutions are summarized in Table 5.

Images from UQ Frame and Arvalis LITERAL additional datasets were larger than the 1,024 × 1,024-px image size used in the challenges. For the GWC_2020 solutions, these larger images were split into a set of 1,024 × 1,024-px patches with a 22% overlap. Models were then applied on each patch and predictions were merged with a Non-Max Suppression algorithm, using the same IoU value used within the model (0.92 for GWC_2020 and 0.7 for the baseline Faster-RCNN). For the GWC_2021 solutions, they were designed to automatically adapt to images with higher resolution thanks to the use of an adaptive pooling and convolution layers.

## Results and Discussion

### The GWC_2020 and GWC_2021 winning solutions

The 3 best solutions provided average accuracy values very close together while largely beating the baseline solution (Table 5). All winners used standard existing open-source architectures, such as EfficientDet, Faster-RCNN, Yolo-v5, and Yolo-v3, without any specific domain adaptation module (Table 5). This indicates that several architectures can generalize to unseen datasets. However, Yolo-v5 was most frequently used during GWC_2021 while it was forbidden during GWC_2020 due to a license conflict with the 2020 competition’s rules.

**Table 5.** Summary of the winning solutions.

	Rank	Solution name	Domain data augmentation	Architecture	Ensemble approach	Challenge score	Comments
Baseline		Madec	No	Faster-RCNN	No	AA_2021_ = 0.474	[13]
GWC_2020	1	DungNB	Mixup and custom mosaic	EfficientDet and Faster-RCNN	Random subsampling	AA_2020_= 0.690	Link to solution
	2	OverFeat	Mixup and cutmix	Efficientdet	Random subsampling	AA_**2020**_= 0.688	Link to solution. Rank first with AA_2021_
	3	Javu	Mixup	YoloV3	No	*AA*_2020_= 0.684	Link to solution
GWC_2021	1	RandomTeam	Mosaic	Yolov5	Domain subsampling	AA_**2021**_= 0.700	Link to solution
	2	David_jeon	Mosaic and cutmix	Yolov5	No	AA_2021_= 0.695	Model is applied on 1,600-px images. Link to solution
	3	SMART	Cutmix	Yolov4	Yes	AA_2021_= 0.695	A network is jointly trained to improve image quality. [44]

Part of the improved performances comes from test-time augmentation combined with the weighted boxes fusion [29] that was used on the 6 winning solutions. Winners used several data augmentation techniques such as mixup [30] and mosaic augmentation [31]. However, it is difficult to assess the effect of specific data augmentation techniques on the robustness, maybe due to an interaction between the model architecture and the data augmentation algorithm. This issue is further discussed in Section S3. All the winning solutions used pseudo-labeling [32], where predicted labels on the validation dataset are called “pseudo-labeled” data and then the model is fine-tuned with a mix of training data and pseudo-labeled data. Further, an ensemble approach was proposed by 5 of the 6 winners, where several models were trained on different subsampling of the training dataset, and their solutions were fused into a single solution. Despite being widely used for data competition, these techniques are not standard in plant phenotyping.

Additional strategies were developed during the competition. First, the participants optimized a few hyperparameters including the score threshold, the IoU threshold, and the image size. None of the winning solutions used the same set of hyperparameters as the proposed baseline solution. For instance, the second winner of GWC_2021 (david_jeon) upsampled the images to 1,600 px before prediction, which could help detect small wheat heads. Another popular approach during the GWC_2020 was to generate more diversity from the training dataset based on “jigsaw puzzle” techniques. Given that some images were cropped from an original larger one, competitors have generated new images by assembling patches cropped randomly in the original images, instead of using a regular grid as in the baseline solution preprocessing step [14].

Surprisingly, the winning solutions did not used domain-adaptation approaches with advanced data augmentation strategies, domain-adversarial training, or new model architectures that could solve the difficulty of overlapping heads and small heads. We hope that the GWHD 2021 can provide a reliable database for the plant phenotyping community.

In the following, we selected 3 solutions to investigate more deeply the performances: the baseline solution (Faster-RCNN), the RandomTeam solution ranked first in the GWC_2021, and the OverFeat solution from the GWC_2020 that ranked first using AA_2021_ while being second using AA_2020_.

### Challenges solved most of the FPs but still miss small wheat heads

**Fig. 3.**
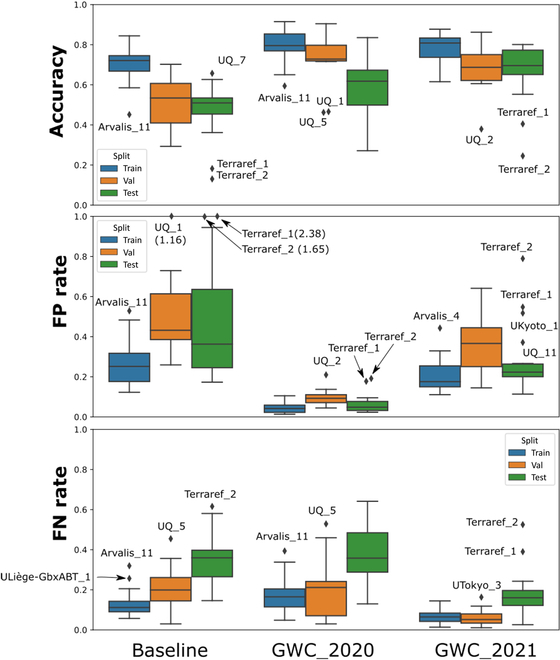
Detailed performance of the best solutions against the baseline. Extreme domain results are indicated by text. Top: Accuracy per domain; center: FPR; bottom: FNR.

The performances measured with the average accuracy (AA_2021_) are heterogeneous between sessions while the ranking of AA_2021_ across training, validation, and test datasets is similar for all the 3 solutions considered here (Fig. 3). It appears that the level of complexity of an acquisition session depends on its inner characteristics and all the solutions experienced difficulties on the same sessions. However, the GWC_2021 always beats the other solutions except for Terraref_2 and NAU_3 sessions. Conversely, the baseline solution has always lower AA_2021_ values than the 2 winning solutions. The GWC_2020 solution show generally AA_2021_ values lower than those of GWC_21, except for few sessions including Terraref 2 and NAU 3.

While the results just discussed correspond to those obtained over the test dataset, it is interesting to compare them to those of the training and validation datasets: it provides some insight on the possible domain shifts and model robustness. The AA_2021_ values of the training dataset always get the best accuracy as expected. This is also observed for the FPR and false negative rate (FNR) that are lower than those of the validation and test datasets (Fig. 3). The validation dataset used for hyperparameter optimization and pseudo-labeling has AA_2021_ values in between the training and the test datasets. The domain shift evaluated by the difference in AA_2021_ between the training and test datasets is reduced for GWC_2021, but important for the 2 other solutions: the GWC_2021 solution appears therefore more robust than the baseline and GWC_2020 models.

The better AA_2021_ obtained for GWC_2021 can be mostly attributed to a reduction of FNs (Fig. 3, bottom): GWC_2021 has lower FNR values across all the sessions. The GWC_2020 and baseline solutions have similar FNRs. Conversely, the GWC_2020 has the smallest false positive rates (FPRs), while the baseline solution shows higher values of FPR (Fig. 3, middle). The GWC_2021 solution shows intermediate values of FPRs. Combining the GWC_2021 and GWC_2020 solutions could be a possible pertinent solution when selecting the bounding boxes to be kept.

Part of the low performance comes from the specificity of certain sessions, possibly explained by several factors: (a) the low spatial resolution of the images explains the degraded performances for Arvalis_4; (b) the combination of wheat head bending, and wheat head awns explain the difficulty of Arvalis_11, UQ_5, and UQ_7; (c) Terraref_1 and Terraref_2 suffer from intense illumination resulting in stark contrasts in the images; (d) for some sessions (Terraref_1, Terraref_2, UQ_1, and Ukyoto_1), a portion of the wheat heads are partly inside the stalk, despite the majority of the wheat heads having emerged; (e) finally, some sessions such as UQ_1 and UQ_2 contain many “empty” images that artificially lowers the accuracy due to the impact of only one error on the score (0 or 1).

**Fig. 4.**
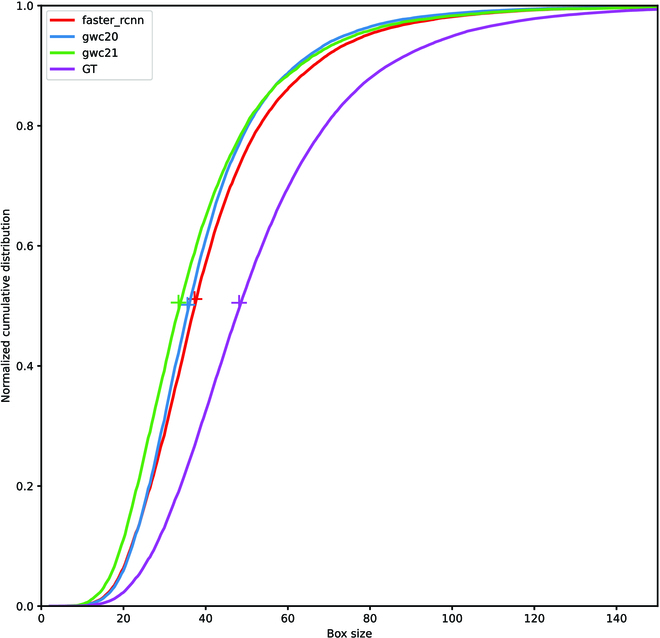
Cumulated frequency of the size of the missing bounding boxes compared to the distribution of the size of all the labeled bounding boxes from the test set (GT).

The FNRs indicate the number of heads that were not detected by the solution. Even with the best solution represented by GWC_2021, all the FNRs are larger than 0.10, except on 4 sessions. This is still a strong limitation for an accurate detection of the heads, explaining why AA_2021_ reaches values ranging from 0.2 to 0.8, with an average close to 0.7. The distribution of the size of the missing boxes (Fig. 4) for all the solutions show that they generally correspond to smaller heads that are more difficult to detect. The average size of the missed heads is about 35% smaller than the average of all heads. Nevertheless, the GWC_2021 solution detects twice as many small wheat heads as compared to the 2 other solutions (Fig. 4) while it is still missing some of the smaller ones.

**Fig. 5.**
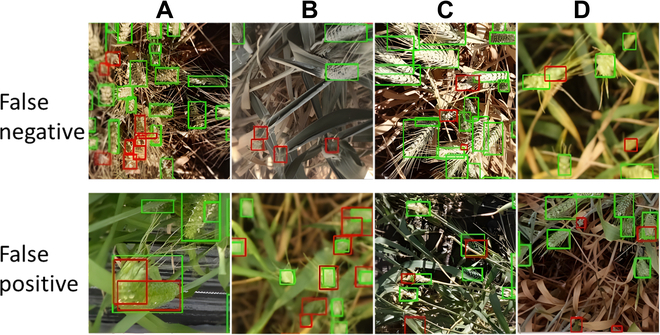
FNs and FPs from GWC_2021 illustrated over a random sample images of the test dataset. Labeled heads are indicated by green bounding boxes, while FNs and FPs are indicated by red bounding boxes. (A) to (D) represent different example images.

The missing heads (FN) are mostly underdeveloped or hidden wheat heads (Fig. 5, top line). Adjusting the IoU threshold may decrease the rate of missing heads. However, improving the detection of smaller wheat heads may increase the FPR, i.e., detecting nonexistent heads. Some of the missed wheat heads may be labeled as nonwheat heads by operators.

The FPs are generally small bounding boxes containing features similar to actual heads (Fig. 5, bottom line), as illustrated by Fig. 5A (False positive) where a curved wheat head is detected twice. More rarely, FP cases are observed (Fig. 5B and C) where leaf parts are mistaken for heads, possibly due to illumination. In addition to better models, using a rigorous acquisition protocol could lower the number of FP.

The balance between FP and FN is tricky: the GWC_2021 reduces the number of FNR at the expense of an increase of FPR, while GWC_2020 keeps low FPR at the expense of a higher FNR. A solution that would reduce both FNR and FPR is still expected, while the balance between both terms is critical when counting heads to estimate the head density.

The winning solutions for GWC_2020 and GWC_2021 use several common features, including (a) a mosaic data augmentation (Section S2), (b) a pseudo-labeling process applied during the inference test time augmentation, (c) the weighted fusion of boxes, and (d) ensemble prediction based on running several models concurrently. The main differences are related to the architecture, with Yolov5 (GWC_2021) instead of EfficientDet (GWC_2020), and the split used to train the several concurrent models. In GWC_2021, the selection is made at the session level instead of the image level, explaining why the various networks used during inference have more contrasted performance. However, luck may have also played some role in the final performance, considering the still limited size of the training, validation, and test datasets used and the stochastic process when training the model.

### Performances for head counting

**Fig. 6.**
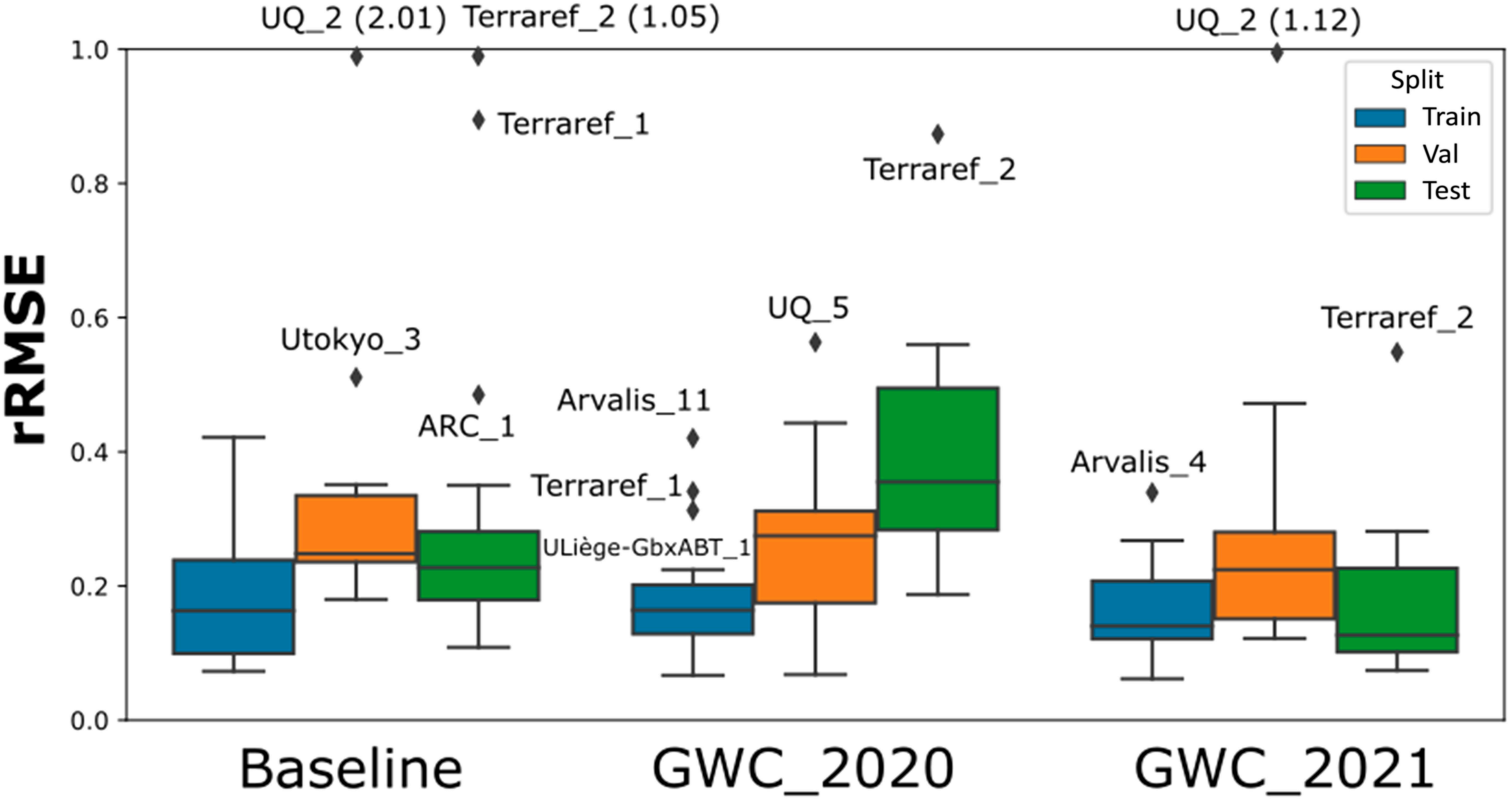
Distribution of the rRMSE per session grouped per dataset split (Train, Validation, and Test) and solutions (Baseline, GWC_2020, and GWC_2021). The middle gray line indicates the median and the blue box the 25% to 75% quantiles. The whiskers extend to the most extreme (1% to 99% assuming a normal distribution), while the diamonds correspond to outliers. The diamonds at the top indicates outliers larger than rRMSE = 1, the rRMSE value being indicated in parentheses.

The rRMSE of head counts was calculated for each session for the training, validation, and test splits. Results show that the rRMSE varies widely across sessions, from 0.66 to 2.15 (Table S2 and Fig. 6). A more detailed inspection of the rRMSE as a function of the solution and the dataset split (Fig. 6) shows that, as expected, the training dataset always has the lowest rRMSE values with no outliers except for GWC_2020. This illustrates that all the solutions are capable to learn the specificities of the several training sessions with only small differences across the 3 models. The performances on the validation dataset used for hyperparameter tuning and pseudo-labeling degrade significantly, with large outliers for the baseline and GWC_2021 solutions. The UQ_2 session from the validation dataset shows the largest outliers for the baseline and GWC_2021 solutions. For the test dataset, the variability between solutions and sessions is still large (Fig. 6). The GWC_2021 model presents the lowest rRMSE values and a moderate dispersion between sessions. This is consistent with the detection performances presented earlier for GWC_2021. The baseline model also shows relatively good performances except for 3 outlier sessions including Terraref_2 that shows also large rRMSE values for the 2 other solutions. The GWC_2020 solution presents the worst performances on the test dataset. The outliers are similar to the ones reported in Section S4.2.

**Fig. 7.**
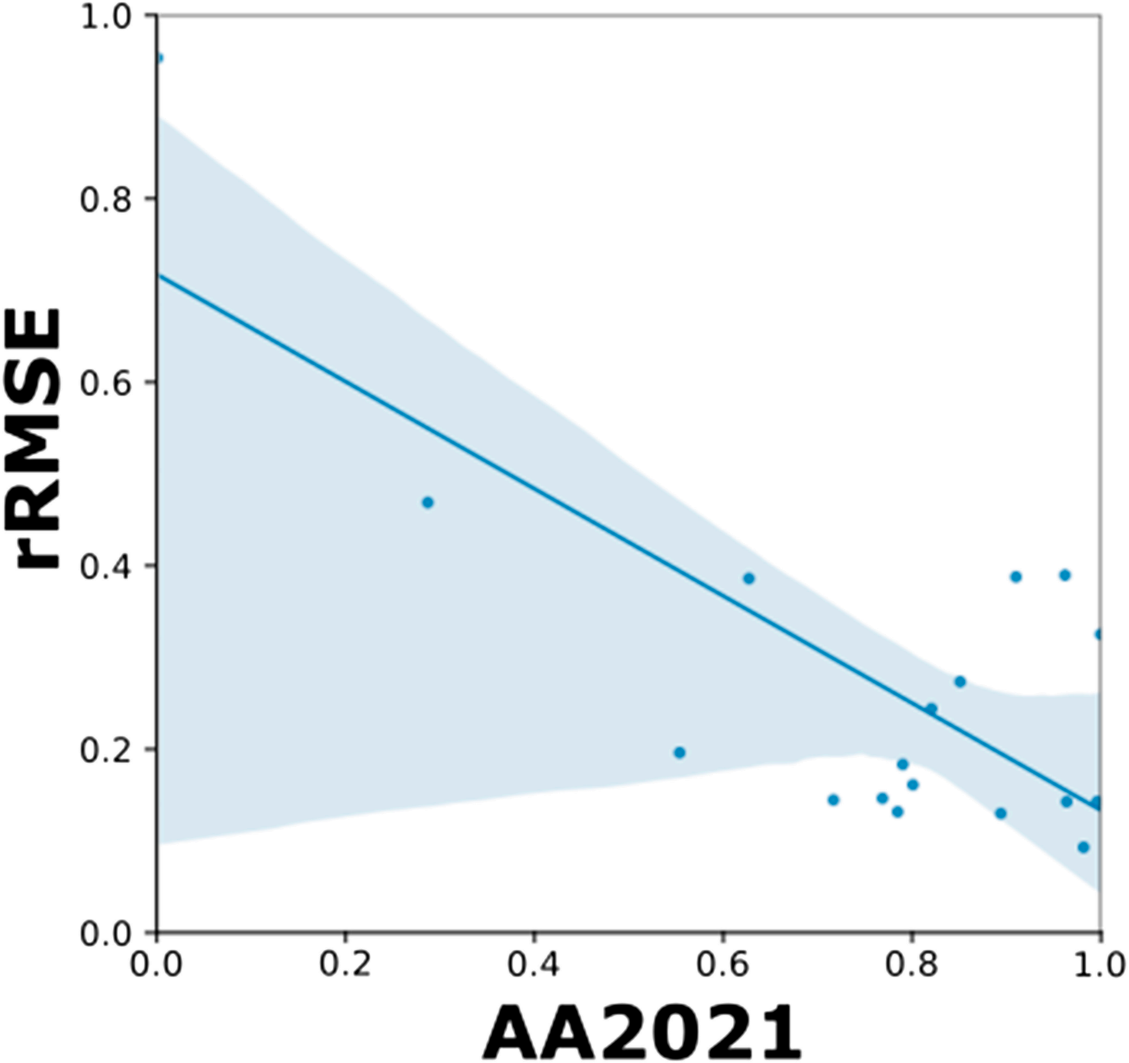
Relationship between AA_2021_ and rRMSE for the test dataset and GWC_2021 solution. Each of the 18 points corresponds to sessions. The confidence interval of 95% is described by the blue line.

The accuracy for head detection is not a good indicator of the counting performances of a solution. While poor accuracy corresponds to high rRMSE for head counting (Fig. 7), no clear relationship is observed between counting performances and the detection accuracy when AA2021 > 0.5 (Fig. 7). For few sessions such as NAU_2 and NAU_3, the rRMSE is below 0.2 despite a good detection accuracy. This is mostly due to the FNs and FPs that are strongly imbalanced (Fig. 3).

The counting performances achieved with GWC_2021 over the test dataset are not as good as reported in other studies such as those by Madec et al. [13] (rRMSE = 0.06) or by Sadeghi-Tehran et al. [33] (rRMSE = 0.11). However, similar performances have been observed for some sessions, including CIMMYT, ARC, KSU, and UQ test datasets. The complexity of generalization over a wide range of conditions corresponding to the several sessions considered in the test dataset makes the detection problem much difficult to solve.

The choice of whether to use localization or regression metrics in applications depends on the specific requirements of the task at hand. Localization is simple to debug and can be used in postprocessing tasks such as organ analysis or robotics, while regression can provide better performance, can be used with smaller networks, and can reduce the need for labeled data. Regression also seems to be more effective at dealing with uncertainty and achieving a good balance of positive and negative examples.

### Comparison with head density measurements in the field

The standard low-throughput method for head density measurements is based on head counting over a relatively small sampling area as described earlier. Two datasets, UQ Frame dataset that includes images from UQ_7 to UQ_11 (Table 3) used in the validation dataset, and Arvalis LITERAL handheld system dataset, acquired concurrently to Arvalis_1 to Arvalis_12 (used for training) but with a different RGB camera. Only the GWC_2021 solution is presented here since we already demonstrated that it outperformed the baseline and GWC_2020 solutions. The performances are evaluated at the microplot level.

**Fig. 8.**
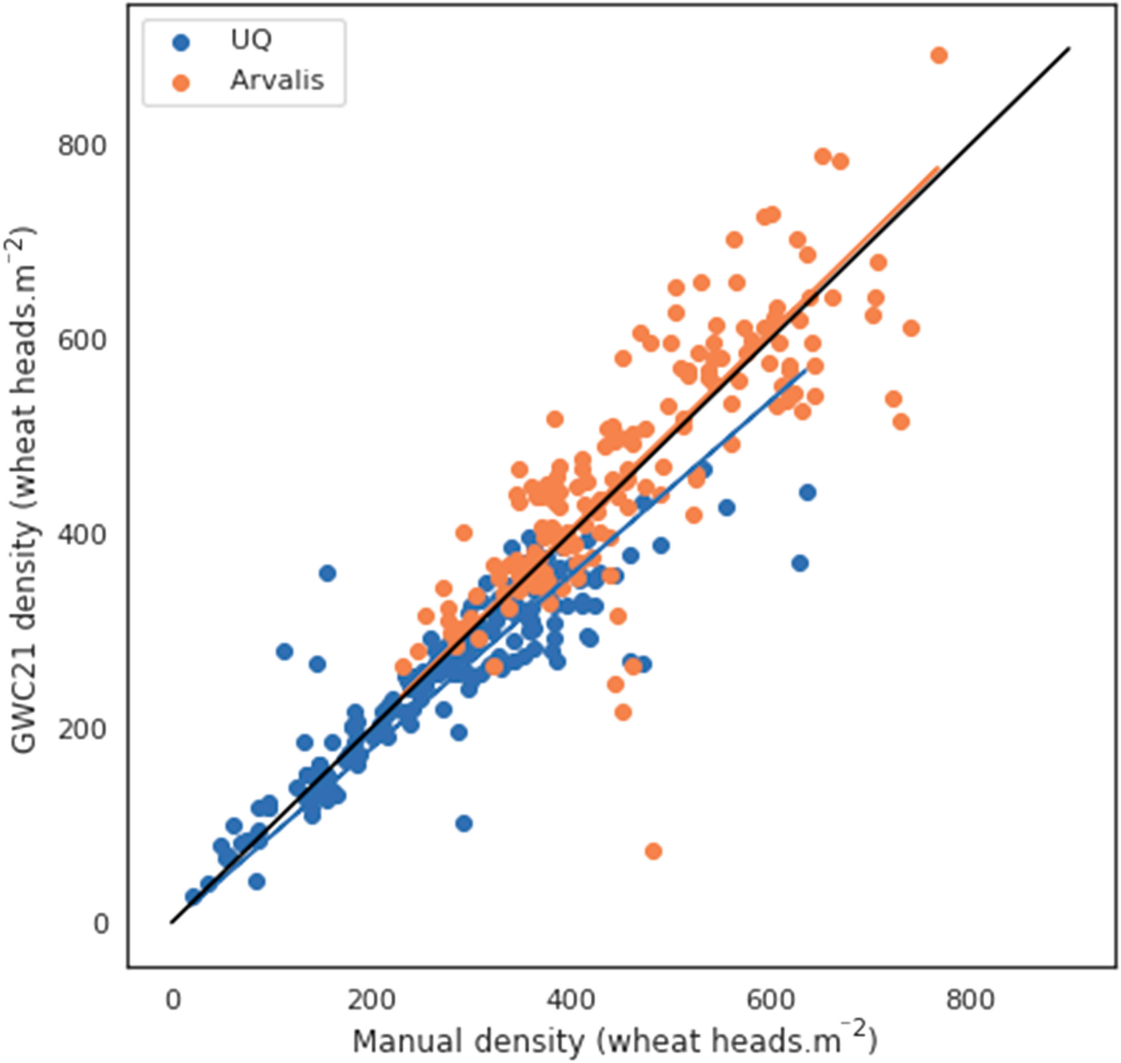
Comparison between the head density measured in the field and that estimated with the GWC_2021 solution from RGB images aggregated at the microplot level. Each point corresponds to a microplot. Blue and red points correspond respectively to UQ and Literal sessions. The black line is the 1:1 line.

There is a good agreement with the head density measured in the field at the plot level (Fig. 8 and Table 6). The discrepancies seem to increase with the head density. Detecting all the heads in the image is more difficult than in the field because of possible occlusions in dense crops. The discrepancies may come from 5 main factors:

1. The spatial sampling: For the Arvalis LITERAL dataset, images and ground samples are not located at the same place, with images covering a larger area (1.4 m^2^) as compared to the area sampled for ground-level head counting (0.7 m^2^).
2. Uncertainties in the area used to compute the density. For the UQ dataset, this uncertainty does not exist since the same frame was used to count heads in the field and to crop the images for head labeling and head prediction. For the Arvalis LITERAL dataset, the area sampled by the image was computed from the focal length of the lens and the pixel size of the complementary metal oxide semiconductor matrix and the knowledge of the distance between the top of the canopy and the camera. This distance was measured with few-centimeter uncertainties. Therefore, the error induced on the area computation should be small, on the order of few percent.
3. Uncertainties in the model to detect heads as seen in the previous section.
4. Differences between heads visible in the RGB image and heads counted in the field. The occluded heads are expected to be more frequent in dense crops (and high head density) as well as when the heads are bending as observed for many genotypes and conditions for the later maturity stages.
5. Errors in head counting by operators in the field that may increase with the head density due to the fatigue of operators, as well as possible occlusions of heads.

**Table 6.** Performances of head density estimation obtained with the GWC_2021 solution.

Datasets	*n*	*R* ^2^	RMSE	rRMSE	Bias	Slope
UQ	185	0.732	56.6	0.205	19.8	0.894
Literal	163	0.633	77.1	0.167	−9.3	1.011
All	348	0.814	67.0	0.185	6.1	0.975

**Fig. 9.**
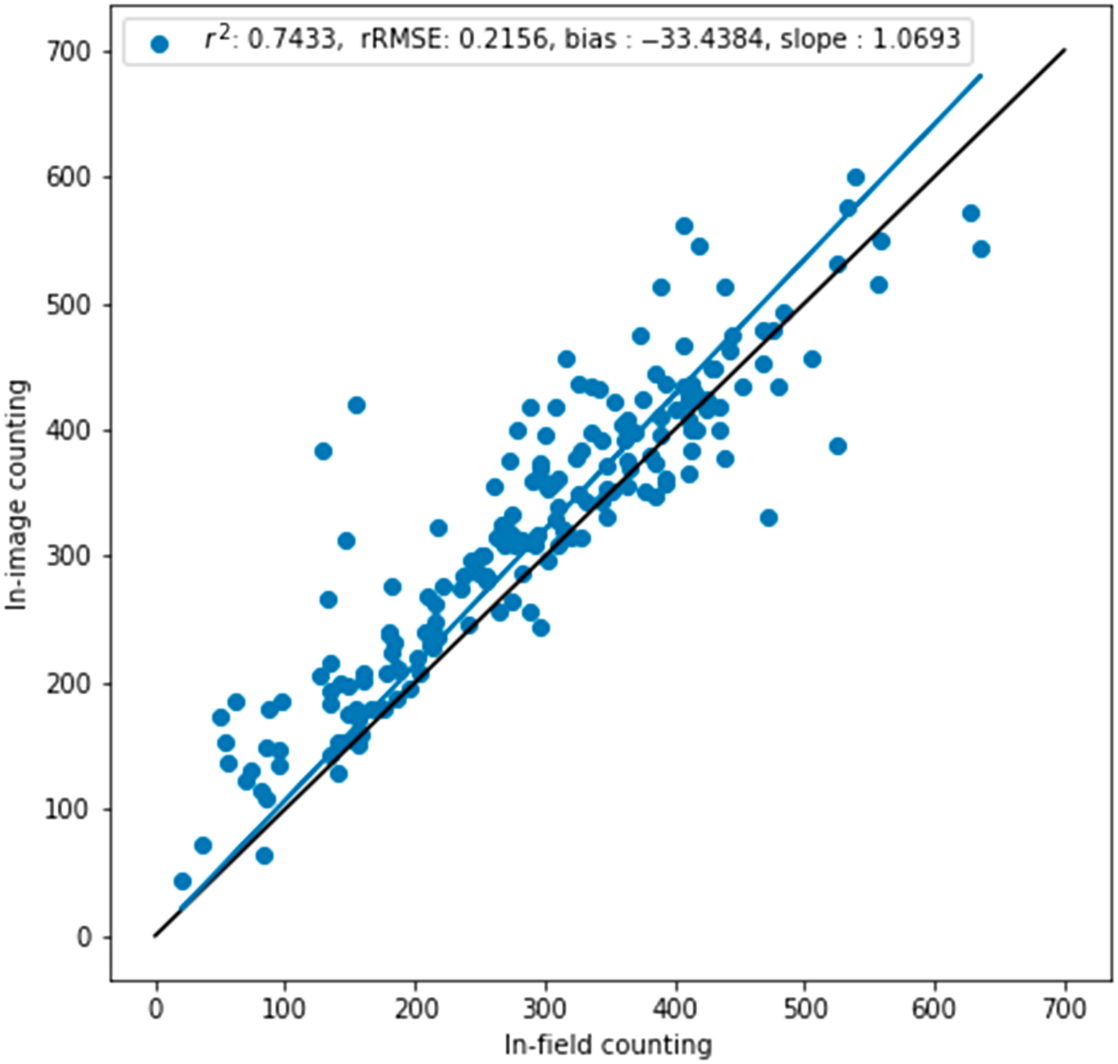
Comparison between the head density measured in the field and that measured on the RGB images by human operators over the same sampling area delimited by a frame. Data coming from the UQ Frame dataset (*n* = 185).

When the head density measured by an operator in the field is compared to that derived from the RGB images labeled by another operator over the same area delimited by the frame used for the UQ dataset, the agreement is loose, with rRMSE = 20.5%, and a slight overestimation of the head density on the images (Fig. 9). We expected that counting on images would underestimate the head count in the field due to unavoidable occlusion as explained earlier. However, the contrary is observed. When counting in the field, the operator did not mark the heads counted and he is likely to double count or miss heads. Conversely, when labeling heads in the images, the operator identifies heads incrementally with a persistent bounding box on each head, which gives a visible indication of which heads have already been counted. Therefore, despite possible head occlusion in the images, we are more confident in the image annotations.

**Fig. 10.**
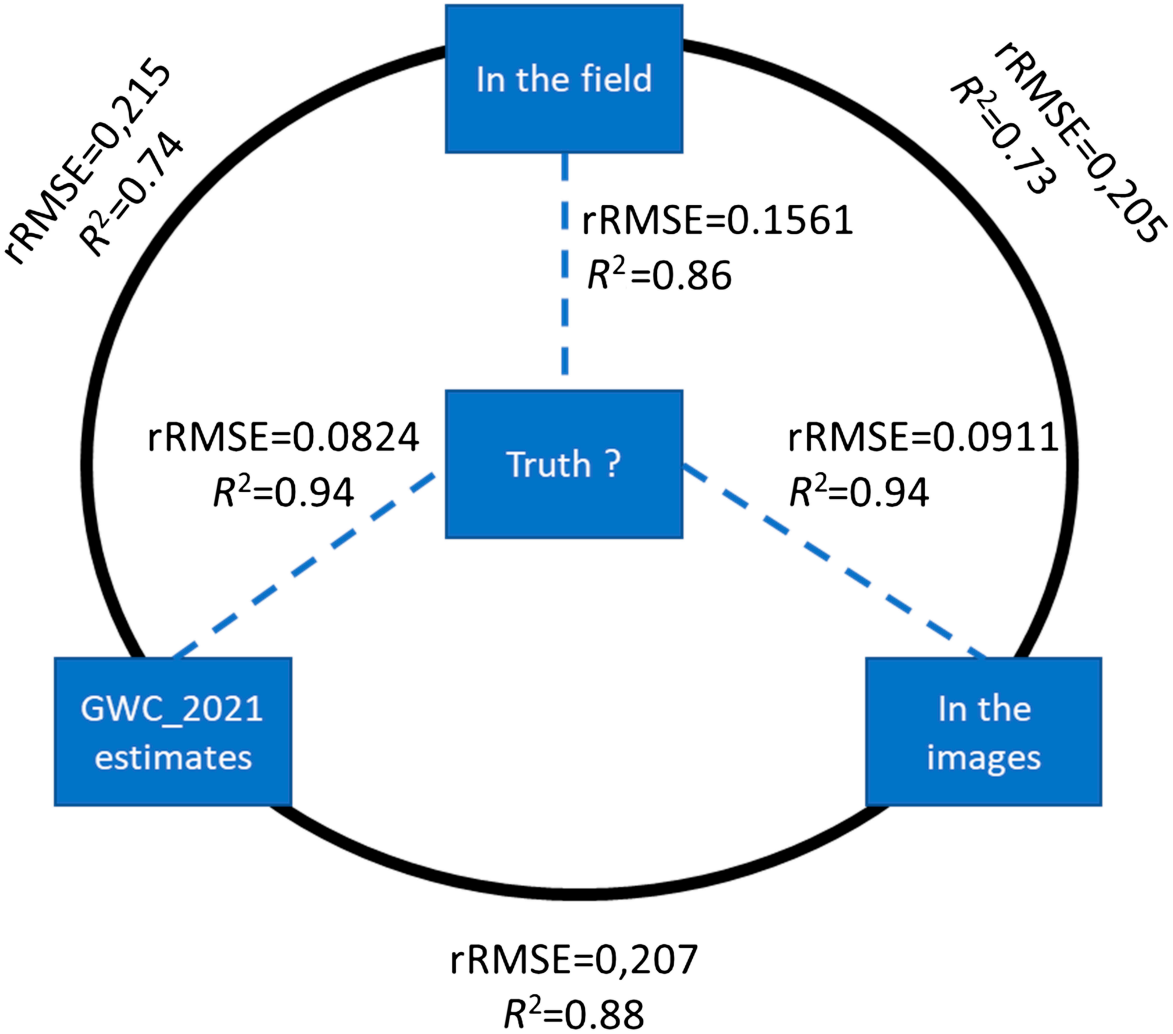
Comparison of the rRMSE and *r*^2^ on head counting on the UQ dataset from the 3 different methods: counting in the field, counting on the images, and head count estimates using the GWC_2021 solution. The unknown ground true number of heads is indicated in the middle of the schema. The statistics (rRMSE and *r*^2^) derived from the triple error colocation error are also indicated in between each method and the central box.

A comparison between the rRMSE obtained over the UQ dataset for head counting in the frames (Fig. 10) shows that the best match is observed between the estimates of the GWC_2021 solution and the head counting in the image. However, the statistics that quantify the agreement between the head count from each approach and the actual head number that is not known could be estimated using the extended triple collocation analysis [34]. This method provides the correlation and the rRMSE from the 3 estimates of the head counts assumed to be independent. Results indicate that head count from the images, using either the labeling by an operator or the estimates from the GWC_2021 solution, are the closest to the true (unknown) value (Fig. 10). Counting the heads in the field appears less accurate, confirming the previous conclusion. Further, counting in the field is tedious and is generally done once by an operator on a limited sampling area. Conversely, counting on images can be repeated by several operators, which should improve the reliability of the result. The triple collocation analysis should ideally be applied to completely independent methods. In the current study, this was not possible as the in-image and GWC_2021 methods were calculated using the same images. It would be worthwhile to conduct this experiment using completely independent methods in the future to confirm the findings.

### Current state of the art in wheat head detection

The design of the competition has evolved between the 2 editions. Additional datasets were used in the GWC_2021, creating more diversity and more images for training. The session, i.e., images acquired with the same system at the same date and location, was recognized as a key structuring factor that defines domains. Greater attention was therefore paid on the balance between sessions in terms of the number of available images. Conversely, in the GWC_2020 edition, Utokyo_1 and Utokyo_2 that contained most of the images in 2020 created an “overfit” artifact. The metrics and the split were also improved in 2021 to better tackle the robustness of the models.

The proposed solutions were not very innovative in terms of model architecture, based on standard 1-step model such as Yolo and EfficientDet. They use specific data augmentation techniques to increase robustness, including mixup, cutmix, and mosaic. Pseudo-labeling was also used to increase the diversity and size of the training dataset. Ensemble approaches were also part of the winning solutions, where several models are fused to achieve a more robust solution. More research in this direction could also led to improvement in generalization for plant phenotyping. These several elements of the winning solutions can be applied for other problems including additional traits and crops. It however requires access to a minimal set of metadata corresponding to a session that defines a domain [14,27].

The GWC_2021 solution is 14 times faster to compute compared to GWC_2020 with less than 1 s per image on computers equipped with a GeForce 1080 GTX or a GeForce 3090 RTX video card. The 3 winning models of both editions were made open-source and can therefore be used by the plant phenotyping community. The corresponding solutions are available on Github (https://github.com/ksnxr/GWC_solution).

Some progress was observed on robustness in the GWC_2021 winning solution as compared to the baseline and GWC_2020 solutions both for head detection and counting. Further, we demonstrated that these techniques based on high-resolution imagery were at least as reliable as the standard approach of low-throughput head counting in the field by an operator: it reduces systematic errors made by the operator and allows to increase the size of the sample used to compute the average head density at the microplot level. Further, the use of images makes the counting process more traceable as compared to counting directly in the field. Nonetheless, our study demonstrates that for well-defined conditions of acquisition, GWC_2021 can be used as a replacement of manual measurement and is already used operationally both at INRAe and Arvalis. However, performances on sessions with accuracy lower than 0.8 for detection and rRMSE larger than 0.10 for counting indicate that significant improvement is still required for the models to identify small differences between genotypes or modalities. A better understanding of the impact of the acquisition system (i.e., camera type, setting, and resolution) could improve both image normalization pre-processing and use of efficient data augmentation techniques. Bounding boxes to identify the heads may be also replaced efficiently by point identification to reduce time for labeling. Further, the problems related to the score threshold and IoU are simplified in this case. Finally, at least for head counting, regression models similar to Tasselnetv2 [26] and its extension Tassenetv3 [35] or the ones used for ACID [36] may also be valid, as previous studies [37] already demonstrate superiority of regression for GWHD 2020. The main challenge for regression-based counting models will be to maintain performance across diverse and untested domains, which will be explored in future work. Further, a potential direction of future research would be to generate synthetic wheat heads to increase the diversity of the data. The use of Generative Adversarial Network or Style Transfer may be a promising solution for extended and diverse training dataset.

Solutions based on new architectures focusing on small objects that are more difficult to detect still need to be explored but may only provide incremental improvements. In comparison, robust algorithms such as GDRO [38] and Deep CORAL [39] should also be investigated in order to improve the generalization power of the model. Further, the information of the domains/sessions was not intensively exploited during the 2 competitions, although it is expected to increase the robustness of the algorithms. However, the format of a challenge is perhaps not optimal for such approaches where an ensemble approach could be developed based on a series of models trained, validated, and tested on several splits using a multifold cross validation approach. In this case, keeping a private split for the competition will reduce the size of the training and validation datasets.

## Conclusion

The Global Wheat Challenge 2020 and 2021 were important steps toward a robust solution to wheat head detection from high-resolution RGB imagery. It complements similar initiatives focusing on plant seedling classification [19], plant pathology classification [40], the agriculture-vision challenge [21], or plant species recognition [41,42]. The Global Wheat Challenge 2020 and 2021 attracted a lot of attention to a central problem in plant phenotyping and contributed to expose the question to a broader community, including that specialized in image processing based on artificial intelligence algorithms. It is also a unique dataset in terms of the diversity of in-field situations that has been also used as a case study to explore the domain shift problem in the framework of the WILDS initiative [43].

The results of the Global Wheat Challenges (2020 and 2021) provide a comprehensive overview of the current state of the art in the field of wheat detection and density estimation. The competition offers valuable insights into the challenges and opportunities in this area of research and highlights the need for improved generalization across different domains, as well as the importance of developing new techniques for plant segmentation, organ segmentation, and growth stage assessment. By presenting a large ensemble of possible solutions, the competition provides a valuable resource for researchers and practitioners in this field.

## Data Availability

The GWHD 2021 can be downloaded here: https://zenodo.org/record/5092309#.YrvsTBXP2Uk

## Supplementary Materials

Supplementary 1 Sections S1 to S4 Figs. S1 to S2 Tables S1 to S3 References [45,46]
