# Supplementary material for: Global Wheat Head Detection Challenges: Winning Models and Application for Head Counting
Source: Plant Phenomics. 2023 Jun 26;5:0059. doi: 10.34133/plantphenomics.0059 (PMC10795497; doi:10.34133/plantphenomics.0059)
Supplement: Supplementary 1 — Sections S1 to S4 Figs. S1 to S2 Tables S1 to S3 References [45,46] [file plantphenomics.0059.f1.docx]

Supplementary Materials

Table S1: The GWHD_2021 dataset composed of sessions groups by location: Red = Europe, Blue = North America, Yellow = Asia, Green= Oceania, Orange=Africa. The development stages are indicated: 1=post-flowering; 2=grain filling; 3=grain ripening.

| Session name | owner | country | location | year | Platform | Development stage | Number of images | Number of head |
| --- | --- | --- | --- | --- | --- | --- | --- | --- |
| Ethz_1 | ETHZ | Switzerland | Usask | 2018 | Spidercam | 2 | 747 | 49603 |
| Rres_1 | Rothamsted | UK | Rothamsted | 2015 | Gantry | 2 - 3 | 432 | 19210 |
| ULiège-GxABT_1 | Uliège/Gembloux | Belgium | Gembloux | 2020 | Cart | 3 | 30 | 1847 |
| NMBU_1 | NMBU | Norway | NMBU | 2020 | Cart | 2 | 82 | 7345 |
| NMBU_2 | NMBU | Norway | NMBU | 2020 | Cart | 3 | 98 | 5211 |
| Arvalis_1 | Arvalis | France | Gréoux | 2018 | Handheld | 1 | 66 | 2935 |
| Arvalis_2 | Arvalis | France | Gréoux | 2018 | Handheld | 2 | 401 | 21003 |
| Arvalis_3 | Arvalis | France | Gréoux | 2018 | Handheld | 2 - 3 | 588 | 21893 |
| Arvalis_4 | Arvalis | France | Gréoux | 2019 | Handheld | 2 | 204 | 4270 |
| Arvalis_5 | Arvalis | France | VLB* | 2019 | Handheld | 2 | 448 | 8180 |
| Arvalis_6 | Arvalis | France | VSC* | 2019 | Handheld | 2 - 3 | 160 | 8698 |
| Arvalis_7 | Arvalis | France | VLB* | 2019 | Handheld | 2 - 3 | 24 | 1247 |
| Arvalis_8 | Arvalis | France | VLB* | 2019 | Handheld | 2 - 3 | 20 | 1062 |
| Arvalis_9 | Arvalis | France | VLB* | 2020 | Handheld | 3 | 32 | 1894 |
| Arvalis_10 | Arvalis | France | Mons | 2020 | Handheld | 2 | 60 | 1563 |
| Arvalis_11 | Arvalis | France | VLB* | 2020 | Handheld | 2 | 60 | 2818 |
| Arvalis_12 | Arvalis | France | Gréoux | 2020 | Handheld | 2 | 29 | 1277 |
| Inrae_1 | INRAe | France | Toulouse | 2019 | Handheld | 2 - 3 | 176 | 3634 |
| Usask_1 | Univ. Saskatchewan | Canada | Saskatchewan | 2018 | Tractor | 2 - 3 | 200 | 5985 |
| KSU_1 | Kansas State Univ | USA | KSU | 2016 | Tractor | 1 | 100 | 6435 |
| KSU_2 | Kansas State Univ. | USA | KSU | 2017 | Tractor | 1 | 100 | 5302 |
| KSU_3 | Kansas State Univ. | USA | KSU | 2017 | Tractor | 2 | 95 | 5217 |
| KSU_4 | Kansas State Univ. | USA | KSU | 2017 | Tractor | 3 | 60 | 3285 |
| Terraref_1 | TERRA-REF | USA | Maricopa, AZ | 2020 | Gantry | 3 | 144 | 3360 |
| Terraref_2 | TERRA-REF | USA | Maricopa, AZ | 2020 | Gantry | 2 | 106 | 1274 |
| CIMMYT_1 | CIMMYT | Mexico | Ciudad Obregon | 2020 | Cart | 1 | 69 | 2843 |
| CIMMYT_2 | CIMMYT | Mexico | Ciudad Obregon | 2020 | Cart | 1 | 77 | 2771 |
| CIMMYT_3 | CIMMYT | Mexico | Ciudad Obregon | 2020 | Cart | 1 | 60 | 1561 |
| Utokyo_1 | UTokyo | Japan | NARO-Tsukuba | 2018 | Cart | 3 | 538 | 14185 |
| Utokyo_2 | UTokyo | Japan | NARO-Tsukuba | 2018 | Cart | 3 | 456 | 13010 |
| Utokyo_3 | UTokyo | Japan | NARO-Hokkaido |  | Handheld | 1-2-3 | 120 | 3085 |
| Ukyoto_1 | UKyoto | Japan | Kyoto | 2020 | Handheld | 1 | 60 | 2670 |
| NAU_1 | NAU | China | Baima | n.a | Handheld | 1 | 20 | 1240 |
| NAU_2 | NAU | China | Baima | 2020 | Cart | 1 | 100 | 4918 |
| NAU_3 | NAU | China | Baima | 2020 | Cart | 2 | 100 | 4596 |
| UQ_1 | UQueensland | Australia | Gatton | 2015 | Handheld | 1 | 22 | 640 |
| UQ_2 | UQueensland | Australia | Gatton | 2015 | Handheld | 1 | 16 | 39 |
| UQ_3 | UQueensland | Australia | Gatton | 2015 | Handheld | 2 | 14 | 297 |
| UQ_4 | UQueensland | Australia | Gatton | 2015 | Handheld | 2 | 30 | 1039 |
| UQ_5 | UQueensland | Australia | Gatton | 2015 | Handheld | 2 - 3 | 30 | 3680 |
| UQ_6 | UQueensland | Australia | Gatton | 2015 | Handheld | 2 - 3 | 30 | 1147 |
| UQ_7 | UQueensland | Australia | Gatton | 2020 | Handheld | 3 | 17 | 1335 |
| UQ_8 | UQueensland | Australia | McAllister | 2020 | Handheld | 3 | 41 | 4835 |
| UQ_9 | UQueensland | Australia | Brookstead | 2020 | Handheld | 2 - 3 | 33 | 2886 |
| UQ_10 | UQueensland | Australia | Gatton | 2020 | Handheld | 2 - 3 | 53 | 8629 |
| UQ_11 | UQueensland | Australia | Gatton | 2020 | Handheld | 1 | 42 | 4345 |
| ARC_1 | ARC | Sudan | Wad Medani | 2021 | Handheld | 2 | 30 | 888 |
|  |  |  |  |  |  | Total | 6515 | 275187 |

Table S2: rRMSE for the three algorithms on all domains. Colors indicate the rRMSE value: green is the lowest and red the largest

| Split | Sessions | rRMSE | | |
| --- | --- | --- | --- | --- |
|  |  | Baseline | GWC_2020 | GWC_2021 |
| Training | Arvalis_1 | 0.23 | 0.15 | 0.22 |
|  | Arvalis_10 | 0.37 | 0.16 | 0.27 |
|  | Arvalis_11 | 0.25 | 0.42 | 0.17 |
|  | Arvalis_12 | 0.40 | 0.18 | 0.25 |
|  | Arvalis_2 | 0.14 | 0.14 | 0.15 |
|  | Arvalis_3 | 0.12 | 0.11 | 0.13 |
|  | Arvalis_4 | 0.42 | 0.12 | 0.34 |
|  | Arvalis_5 | 0.22 | 0.10 | 0.25 |
|  | Arvalis_6 | 0.08 | 0.16 | 0.13 |
|  | Arvalis_7 | 0.20 | 0.21 | 0.12 |
|  | Arvalis_8 | 0.17 | 0.18 | 0.09 |
|  | Arvalis_9 | 0.15 | 0.19 | 0.06 |
|  | ETHZ_1 | 0.07 | 0.16 | 0.13 |
|  | Inrae_1 | 0.15 | 0.08 | 0.19 |
|  | NMBU_1 | 0.08 | 0.31 | 0.10 |
|  | NMBU_2 | 0.24 | 0.22 | 0.12 |
|  | Rres_1 | 0.09 | 0.07 | 0.15 |
|  | ULiege-GxABT_1 | 0.09 | 0.34 | 0.10 |
| Validation | NAU_1 | 0.24 | 0.29 | 0.47 |
|  | UQ_1 | 0.32 | 0.44 | 0.24 |
|  | UQ_2 | 2.02 | 0.27 | 1.13 |
|  | UQ_3 | 0.25 | 0.33 | 0.21 |
|  | UQ_4 | 0.26 | 0.28 | 0.12 |
|  | UQ_5 | 0.35 | 0.56 | 0.14 |
|  | UQ_6 | 0.18 | 0.29 | 0.22 |
|  | Usask_1 | 0.25 | 0.10 | 0.25 |
|  | Utokyo_1 | 0.19 | 0.07 | 0.15 |
|  | Utokyo_2 | 0.23 | 0.07 | 0.16 |
|  | Utokyo_3 | 0.51 | 0.25 | 0.31 |
| Test | ARC_1 | 0.49 | 0.25 | 0.11 |
|  | CIMMYT_1 | 0.18 | 0.37 | 0.12 |
|  | CIMMYT_2 | 0.14 | 0.24 | 0.10 |
|  | CIMMYT_3 | 0.22 | 0.27 | 0.09 |
|  | KSU_1 | 0.27 | 0.36 | 0.07 |
|  | KSU_2 | 0.19 | 0.34 | 0.10 |
|  | KSU_3 | 0.18 | 0.32 | 0.17 |
|  | KSU_4 | 0.20 | 0.35 | 0.10 |
|  | NAU_2 | 0.29 | 0.19 | 0.20 |
|  | NAU_3 | 0.26 | 0.19 | 0.24 |
|  | Terraref_1 | 0.90 | 0.56 | 0.28 |
|  | Terraref_2 | 1.05 | 0.87 | 0.55 |
|  | UQ_10 | 0.17 | 0.46 | 0.16 |
|  | UQ_11 | 0.35 | 0.50 | 0.24 |
|  | UQ_7 | 0.11 | 0.34 | 0.24 |
|  | UQ_8 | 0.23 | 0.52 | 0.10 |
|  | UQ_9 | 0.21 | 0.53 | 0.10 |
|  | Ukyoto_1 | 0.24 | 0.49 | 0.13 |

1. **Limits of GWC_2020 competition design**

The design of a competition is critical to obtain solutions that satisfy its objective. The metric proposed during the Kaggle competition had the drawback of not being an open implementation. Our open re-implementation of the metric reaches similar but not identical scores to those reported by Kaggle. Unfortunately, these small changes in score can lead to severe changes in ranking. The results presented in Figure S1-A reflect how the corrections and the re-implementation could have drastically changed the ranking on the Kaggle annotations.

Domain generalization is a core problem that the Global Wheat Head Dataset aims to solve but the metric chosen for the Kaggle challenge was too heavily influenced by the performance on the largest domain – utokyo_1. The use of weighted accuracy promotes solutions that have more balanced performance. Figure S1-B illustrates that the weighted accuracy is less saturated than the original metric with performance varying between 0.3 and 0.6 while the AA varies between 0.65 and 0.7.

Applying these results would have influenced the final ranking. Overfeat, which has ranked 2nd and DungNB which has ranked 1st would have ranked 1st and 3rd, respectively. Peculiarly, the solution that was ranked 9^th^ would be 2^nd^, and the 3^rd^ place solution would have dropped below a rank of 100. These results demonstrate the robustness of the solutions by Overfeat and DungNB despite the original metric. However, the former metric could have discouraged even more robust solutions from rising to the top. The solution by Overfeat will be studied closely in the paper because it scored significantly higher (+0.03) than the second solution on the weighted accuracy.


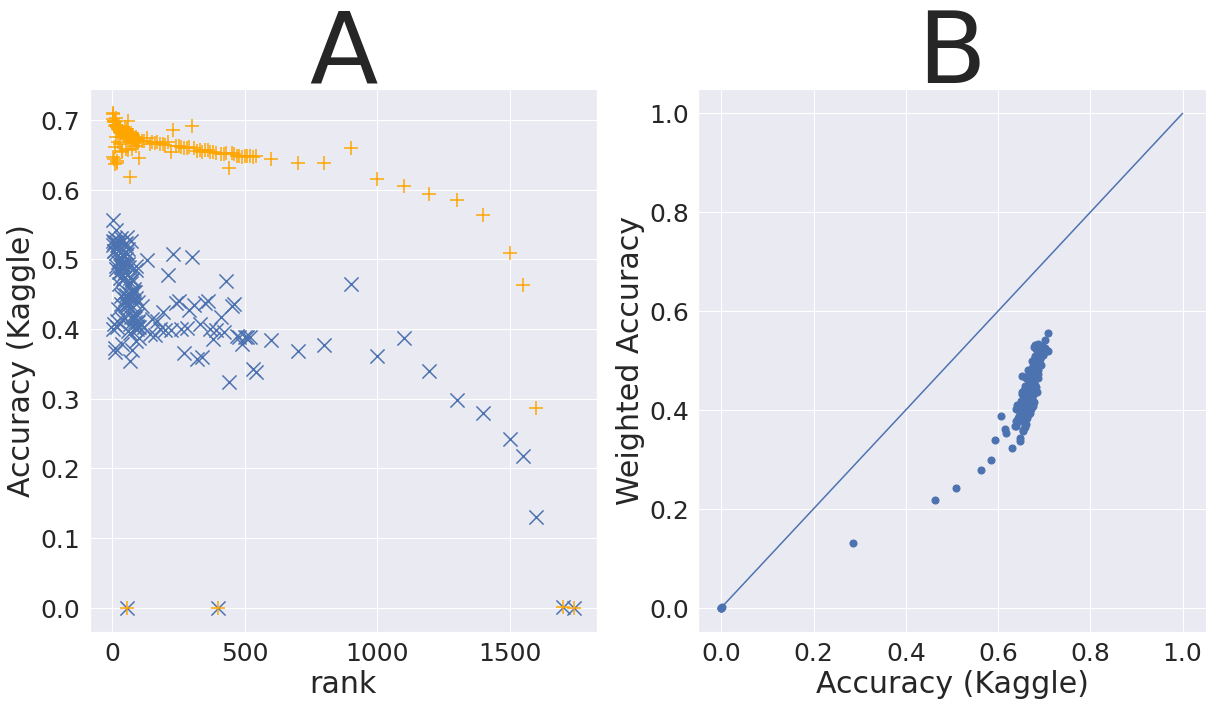


Figure S1: Presentation of the limit of the ranking used during the competition. The x-axis of Part A represents the actual ranking in the Kaggle competition while the y-axis shows the score. The scores with our re-implementation of the metric are shown in orange and the proposed simplified metric are shown in blue. Both are evaluated on the corrected private test set. Part B the score for each solution with metric used during Kaggle against the AA_2021_. Part C presents a simulation of the new ranking based on the new score and limited to the solutions sampled for the analysis.

1. **Data augmentation**

The 20 solutions getting the highest AA_2020_ were reviewed to evaluate the impact of the data augmentation strategies on the model detection performance. About 40 types of augmentation techniques were used, most of them being applied to single images, while three others require several images to be exploited concurrently. The three multi-image augmentation techniques were selected for further analysis. About 15 single-image augmentation techniques were selected to represent the main types of techniques used, while eliminating those that were a priori not pertinent in our context such as RandomSnow or RandomSunFlare. The single-image data augmentation techniques were implemented using a popular image processing library known as Albumentations.

The multi-image augmentation techniques (Mixup, Cutmix), presented in Figure S2, were used for the Overfeat solution (Table 3, Figure 2). Mixup (Zhang *et al.*, 2018) and Cutmix (Yun *et al.*, 2019) both mix 2 images to form an augmented image. While Mixup uses a weighted sum of the pixels from the two images, Cutmix replaces a patch in one image with a randomly cut patch from another image. In this study, the weights for the Mixup operation were set to a constant, 0.5, although it could also be drawn from a random distribution for each image. The Mosaic augmentation technique is an extension of Cutmix and takes a batch of 4 images, randomly scales each image and patches them together into a single image. Because of its close nature to cutmix, it has not been included in the ablation study. The annotations for each image are proportionately scaled and stacked together to form the augmented annotations.

The EfficientDet-D4 [46] object detection model, pretrained on the Microsoft Common Objects in Context (MS-COCO) dataset [9] was selected as the baseline model for evaluating the effects of these data augmentation techniques on the wheat detection task using the GWHD dataset. All data augmentation strategies used in this study were applied only to the training dataset, with a probability of 0.5. Only one augmentation per group of similar operations is selected randomly for each batch.

| 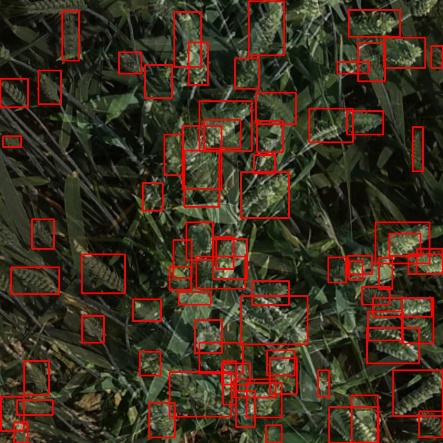 | 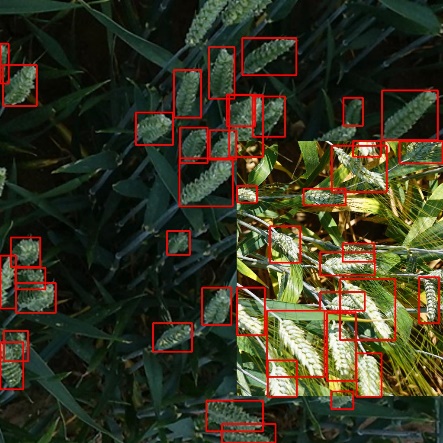 | 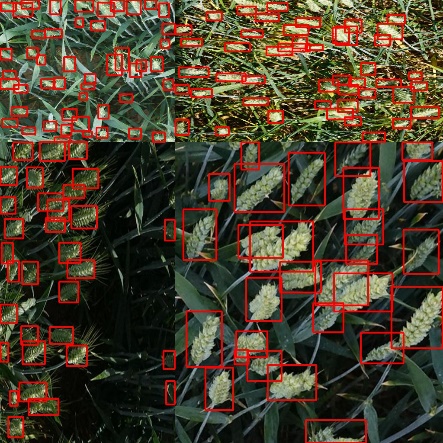 |
| --- | --- | --- |

Figure S2: Presentation of Mixup, Cutmix and Mosaic strategy

1. **Data augmentation and pseudo-labelling performance**

| Modality | A_w_ | A_GWC_2020_ |
| --- | --- | --- |
| Baseline | 0.452787 | 0.654569 |
| Baseline + Cutmix | 0.476214 | 0.659364 |
| Baseline + Mixup | 0.367481 | 0.553144 |
| Data Augmentation (DA) | 0.498065 | 0.704271 |
| DA + Cutmix | 0.501241 | 0.685546 |
| DA + Mixup | 0.437714 | 0.686517 |
| DA + Cutmix + Mixup | 0.358226 | 0.508334 |

Table S3: Ablation study different Data Augmentation strategies

Data augmentation was found to be important for improving the robustness of the trained models (Table S1). The classic data augmentation increases the weighted accuracy by 0.045, almost 10% of the baseline score. Advanced data augmentation techniques such as Cutmix [45] or Mixup [31] do not seem beneficial for the training – while Cutmix alone improves performance by 0.023, it does not add any performance compared to classic data augmentation techniques. An interesting finding is that some data augmentation can decrease the robustness – the use of Mixup always decreases the performance. Our results suggest that while the use of Cutmix or Mixup theoretically increases robustness in the use case of classification, it does not seem to translate for detection. Conclusions are similar when using the same accuracy as in the Kaggle challenge. This result contradicts the popularity of such approaches during the Kaggle competition.

In our experiment, Mixup could be drawn with a probability of 0.5, while it’s reduced to 0.165 in the case of the winning solution. The quality of our classic data augmentation pipeline, which is inspired by the strongest solutions could also explain the results. Our results confirm the importance of data augmentation for robustness but call for more careful exploration when applying usual, typically multi-image, data augmentation techniques. It is particularly important to remember that results on classification tasks may not translate well on detection. The use of bounding boxes may also limit the use of strategies such as Cut, Paste and Mix to increase the diversity of the data.

1. **Organizing an image-based phenotyping**

**4.1 – Basic steps to follow to organize a competition**

We defined 9 steps to organize a successful data competition:

1. **Define the objective of the competition and its goals.** For example, the competition could aim to improve algorithms for detecting wheat head density in order to improve agricultural experimentation.
2. **Identify potential sponsors for the competition.** These could include research institutes, agricultural organizations, and companies involved in agricultural technology.
3. **Select a platform for hosting the competition**, such as Kaggle or AICrowd. Kaggle is a well-established platform that has a large community of data scientists and machine learning experts. It offers a range of tools and resources for organizing and running competitions, but it can be expensive for organizations to run competitions on the platform and it can be difficult for beginners to participate due to the high level of competition and expertise in the community. AICrowd is a newer platform that is focused on supporting machine learning challenges and competitions. It offers similar tools and resources as Kaggle, but it is not as well-known and some users have reported that it can be difficult to use. It can also be self-hosted on platform such as Eval.AI but it has not been tested by the authorts.
4. **Develop a dataset of images of wheat heads and define the labels for the data**, such as head density and location. The dataset should include images from different environments and conditions to test the generalization ability of the algorithms.
5. **Define the rules and evaluation criteria for the competition.** This could include requirements for the algorithms, such as using existing architecture, and metrics for evaluating the submissions, such as accuracy and speed. A specific attention should be brought to the quality of the evaluation metric to avoid overfitting on the competition’s specificities.
6. **Create a communication plan to engage with potential participants** and keep them informed about the competition. This could include regular updates on the competition website and forums, as well as social media outreach.
7. **Once the competition is launched, monitor the submissions and provide support** and guidance to participants as needed. It is important to dedicate one person during the entire duration of the competition.
8. **At the end of the competition, evaluate the submissions and select the winners based** on the evaluation criteria.
9. **Share the results of the competition**, including the winning algorithms and any insights or lessons learned, with the broader community. It can be done through scientific papers but also blog post, posters during conference etc…

**4.2- Pitfalls to avoid**

In the Global Wheat Kaggle Challenge, the organizers encountered three main issues. The first was a problem with the quality of the data, which resulted from differences in the definitions of labels across the datasets and errors in the export tool used to create the dataset. This affected the fairness of the competition and required the organizers to audit the test dataset before the end of the competition in 2020. These issues were solved in 2021. Data quality is a major issue for most competitions.

The choice of the evaluation metric for a data science competition is an important decision that can impact how submissions are judged and ranked. A good evaluation metric should be relevant to the goals of the competition, easy to understand and calculate, and able to differentiate between submissions. However, evaluation metrics can be subject to bias or manipulation, which can lead to unfair comparisons and undermine the integrity of the competition. In the Global Wheat Kaggle Challenge, the common accuracy metric used in 2020 was not appropriate for the generalization purpose of the competition, so it was improved to the domain accuracy metric in 2021.

The third issue was a problem with communication regarding licensing issues. The organizers had asked for solutions to be MIT-compliant, but the winning solution, Yolov5, was issued with a GPL license, which was not compatible with the MIT license. This caused confusion among participants and required the organizers to provide clarification on the forum.

These issues highlight the importance of careful planning and communication in organizing a data science competition.
